# Nutrition transition in Brazilian children under 5 years old from 2006 to 2019

**DOI:** 10.1590/0102-311XEN216622

**Published:** 2023-10-23

**Authors:** Inês Rugani Ribeiro de Castro, Luiz Antonio dos Anjos, Elisa Maria de Aquino Lacerda, Cristiano Siqueira Boccolini, Dayana Rodrigues Farias, Nadya Helena Alves-Santos, Paula Normando, Maiara Brusco de Freitas, Pedro Gomes Andrade, Neilane Bertoni, Raquel Machado Schincaglia, Talita Lelis Berti, Letícia B. Vertulli Carneiro, Gilberto Kac

**Affiliations:** 1 Instituto de Nutrição, Universidade do Estado do Rio de Janeiro, Rio de Janeiro, Brasil.; 2 Departamento de Nutrição Social, Universidade Federal Fluminense, Niterói, Brasil.; 3 Instituto de Nutrição Josué de Castro, Universidade Federal do Rio de Janeiro, Rio de Janeiro, Brasil.; 4 Instituto de Comunicação e Informação Científica e Tecnológica em Saúde, Fundação Oswaldo Cruz, Rio de Janeiro, Brasil.; 5 Instituto de Estudos em Saúde e Biológicas, Universidade Federal do Sul e Sudeste do Pará, Belém, Brasil.; 6 Divisão de Pesquisa Populacional, Instituto Nacional de Câncer José Alencar Gomes da Silva, Rio de Janeiro, Brasil.; 7 Instituto de Estudos em Saúde Coletiva, Universidade Federal do Rio de Janeiro, Rio de Janeiro, Brasil.

**Keywords:** Growth Disorders, Overweight, Anemia, Vitamin A Deficiency, Breastfeeding, Transtornos do Crescimento, Sobrepeso, Anemia, Deficiência de Vitamina A, Aleitamento Materno, Trantornos del Crecimiento, Sobrepeso, Anemia, Deficiencia de Vitamina A, Lactancia Materna

## Abstract

This manuscript aims to report the nutrition transition in Brazilian children under 5 years old from 2006 to 2019. Microdata from the *Brazilian National Survey on Demography and Health of Women and Children* (PNDS 2006) and the *Brazilian National Survey on Child Nutrition* (ENANI-2019) were analyzed. The indicators considered were: micronutrient status (anemia and vitamin A deficiency), anthropometric status (stunting and excessive weight), and breastfeeding practice (exclusive breastfeeding among children < 6 months and continued breastfeeding among children 12-23 months). We also analyzed minimum dietary diversity (MDD), consumption of ultra-processed foods, consumption of meat or eggs, and not consuming fruits or vegetables in children 6-59 months of age only for ENANI-2019. Equiplot charts were generated according to geographic region, maternal schooling level, and maternal race/skin color. From 2006 to 2019, the prevalence rates of anemia and vitamin A deficiency decreased from 20.5% to 10.1% and 17.2% to 6%, respectively. The prevalence of stunting remained at 7%, and excessive weight rates increased from 6% to 10.1%. The prevalence of exclusive breastfeeding among children < 6 months increased from 38.6% to 45.8%, and of continued breastfeeding among children 12-23 months from 34.6% to 43.6%. In 2019, 61.5% of children achieved the MDD, 88.8% consumed ultra-processed foods, 83.1% consumed meat or egg, and 25.7% did not consume fruits or vegetables the day before the survey. Trends of decreased micronutrient deficiencies, increased breastfeeding, and excessive weight rates, as well as reductions in disparities related to geographic region, maternal schooling level, and maternal race/skin color, were observed for most of the indicators.

## Resumo

Buscamos reportar a transição nutricional em crianças brasileiras menores de 5 anos de idade entre 2006 e 2019. Foram analisados microdados da *Pesquisa Nacional de Demografia e Saúde da Criança e da Mulher* (PNDS 2006) e do *Estudo Nacional de Alimentação e Nutrição Infantil* (ENANI-2019). Foram considerados os seguintes indicadores: *status* de micronutrientes (anemia e deficiência de vitamina A), estado nutricional antropométrico (excesso de peso e baixa estatura) e a prática de aleitamento materno (aleitamento materno exclusivo entre crianças < 6 meses e aleitamento materno continuado entre crianças de 12-23 meses). Analisamos a diversidade alimentar mínima (DAM), o consumo de alimentos ultraprocessados, de carne ou ovos e o não consumo de frutas ou hortaliças apenas para o ENANI-2019 em crianças de 6-59 meses de idade. *Equiplots* foram gerados de acordo com a região geográfica, escolaridade e raça/cor da pele maternas. Entre 2006 e 2019, as prevalências de anemia e deficiência de vitamina A diminuíram de 20,5% para 10,1% e de 17,2% para 6%, respectivamente. A prevalência de déficit de estatura manteve-se em 7% e a de excesso de peso aumentou de 6% para 10,1%. A prevalência de aleitamento materno exclusivo entre crianças < 6 meses aumentou de 38,6% para 45,8% e a de aleitamento materno continuado entre crianças de 12-23 meses aumentou de 34,6% para 43,6%. Em 2019, 61,5% das crianças atingiram a DAM, 88,8% consumiram alimentos ultraprocessados, 83,1% consumiram carne ou ovos e 25,7% não consumiram frutas ou hortaliças no dia anterior à pesquisa. Observamos tendências de diminuição das deficiências de micronutrientes, aumento do aleitamento materno e excesso de peso e reduções em disparidades regional, de escolaridade e de raça/cor da pele maternas para a maioria dos indicadores.

## Resumen

Buscamos informar sobre la transición nutricional en niños brasileños menores de 5 años entre 2006 y 2019. Se analizaron microdatos de la *Encuesta Nacional de Demografía y Salud del Niño y de la Mujer* (PNDS 2006) y del *Encuesta Nacional de Alimentación Nutrición Infantil* (ENANI-2019). Se consideraron los siguientes indicadores: estado de micronutrientes (anemia y deficiencia de vitamina A), estado nuricional antropométrico (sobrepeso y baja estatura) y la práctica de la lactancia materna (lactancia materna exclusiva en niños < 6 meses y lactancia materna continua entre niños de 12-23 meses) como indicadores. Analizamos la diversidad dietética mínima (DDM), el consumo de alimentos ultraprocesados, carne o huevos, y el no consumo de frutas o verduras solo para ENANI-2019 en niños de 6-59 meses de edad. Se generaron *equiplots* en función de la región geográfica, la educación y raza/color de la piel de la madre. Entre 2006 y 2019, las prevalencias de anemia y deficiencia de vitamina A disminuyeron del 20,5% al 10,1% y del 17,2% al 6%, respectivamente. La prevalencia del déficit de estatura se mantuvo en el 7 % y la de sobrepeso aumentó del 6% al 10,1%. La prevalencia de lactancia materna exclusiva en niños < 6 aumentó del 38,6% al 45,8% y la de lactancia materna continua entre niños de 12-23 meses aumentó del 34,6% al 43,6%. En 2019, el 61,5% de los niños alcanzaron DDM, el 88,8% consumieron alimentos utraprocesados, el 83,1% consumieron carne o huevos y el 25,7% no consumieron frutas o verduras el día anterior a la encuesta. Observamos tendencias de disminución de las deficiencias de micronutrientes, un aumento de la lactancia materna y sobrepeso y reducciones en las disparidades regionales, de escolaridad y de raza/color de la piel de la madre para la mayoría de los indicadores.

## Introduction

In the last 30 years, the nutritional profile of populations worldwide has transitioned to an increasing prevalence of obesity and a decreasing prevalence of undernutrition in children, adolescents, and adults ^1^ ^,^ ^2^. From 2000 to 2019, the worldwide prevalence of excessive weight among children < 5 years old increased from 4.9% to 5.6% ^3^. Moreover, of children < 5 years old, approximately 22% suffer from stunting, 7.3% from wasting, and at least 50% from micronutrient deficiencies ^4^. The problems caused by the double burden of malnutrition prevail in many populations, with the coexistence of nutritional deficiencies (micronutrient deficiencies, underweight, and childhood stunting and wasting) and overweight/obesity ^5^.

In Brazil, analyses of national surveys demonstrated a massive reduction in stunting, decreasing from 37% (1974-1975) to 7% (2006-2007), and no increase in the prevalence of excessive weight among children < 5 years old (6%) from 1996 to 2006-2007 ^6^ ^,^ ^7^. They also revealed a substantial increase in exclusive breastfeeding among children < 6 months of age, ranging from 4.7% (1986) to 37.4% (2006-2007) ^8^. The *Brazilian National Survey on Demography and Health of Women and Children* carried out in 2006-2007 (PNDS 2006) was the first national study to investigate the prevalence of anemia (20.9%) and vitamin A deficiency (17.4%). Therefore, it was impossible to examine the temporal trends of these outcomes ^9^.

Despite these advances, the complex interplay of social, economic and political determinants of all forms of malnutrition still results in substantial inequalities between subgroups within populations ^10^ ^,^ ^11^. In Brazil, this situation has been aggravated by the dismantling of public policies from 2016-2022 ^12^ ^,^ ^13^ and the increase in food insecurity registered since 2017-2018 ^14^.

To overcome this gap in data on child nutrition based on household surveys, the *Brazilian National Survey on Child Nutrition* (ENANI-2019) was carried out in 2019 ^15^. ENANI-2019 is a national household survey made to guide the creation and reorientation of public policies on feeding, nutrition and health directed to children < 5 years old. This study aimed to examine temporal trends and social and regional inequalities for Brazilian children regarding the prevalence of anemia, vitamin A deficiency, excessive weight, stunting, exclusive breastfeeding among children < 6 months, and continued breastfeeding among children 12-23 months by comparing the results of PNDS 2006 with those of ENANI-2019. This study also aimed to describe dietary quality indicators, available only in ENANI-2019. Together, these analyses present updates on the feeding and nutrition scenario and contribute to understanding the nutritional transition of Brazilian children < 5 years old.

## Methods

We analyzed microdata from two national surveys conducted with children < 5 years old: PNDS 2006 and ENANI-2019. Both were household-based, nationwide surveys with a complex probabilistic cluster sampling design and geographic stratification by macroregions. More details about the sample of these surveys can be accessed in specific publications ^6^ ^,^ ^16^.

Microdata from PNDS 2006 were obtained from the website of the Brazilian Ministry of Health ^17^. This survey targeted women 15-49 years old (respondents) and their children < 5 years old. It had a total sample of 4,817 children from 3,941 households. Such database has missing values for some characteristics of interest, implying variation in sample size depending on the variable under analysis. Implausible values for the indicators body mass index (BMI)-for-age (BAZ < -5 and > 5) and height-for-age (HAZ < -6 and > 6) were excluded. Sample size details are available in the Supplementary Material (https://cadernos.ensp.fiocruz.br/static//arquivo/supl-e002166-22_8833.pdf).

ENANI-2019 surveyed 14,558 children < 5 years old residing in 12,524 households, with the respondent being the child’s mother or caregiver. Missing data were randomly imputed using the “hot deck” or “nearest neighbor” methods ^18^. Thus, the total number of children and households assessed was included in the analyses, except for anthropometric measurements and biochemical markers. For anthropometric nutritional status analyses, children with syndromes that prevented height measurement (length/stature, n = 5) and those for whom it was not possible to calculate the Z-score for BAZ due to the absence of reference curves (n = 192; premature children < 454 days from conception) were excluded ^19^. For the analyses of anemia (n = 8,187) and vitamin A deficiency (n = 8,393), specific sample weights calculated to compensate for nonresponse for these indicators were used ^16^ (Supplementary Material. https://cadernos.ensp.fiocruz.br/static//arquivo/supl-e002166-22_8833.pdf).

### Socioeconomic and demographic indicators

Characterization of the population of children < 5 years old was performed by using the prevalence of socioeconomic and demographic variables available in the two surveys: maternal schooling level, self-reported maternal race/skin color (individual level), participation in the Brazilian Income Transfer Program, sanitary sewage, access to tap water, and internet access (household level).

The maternal schooling level was constructed considering the number of completed years of study, then grouped into 0-7, 8-10, 11, and ≥ 12 years of education. In the ENANI-2019, schooling from another caregiver other than the mother for 622 children (4.3% of the total sample) was considered in the absence of information on maternal schooling. The categories of self-reported maternal race/skin color were: white, black, mixed-race, yellow (Asian origin according to the Brazilian Institute of Geography and Statistics, e.g., Japanese, Chinese and Korean), and indigenous. The Brazilian Income Transfer Program receipt was measured considering the existence of a resident in the household who was a beneficiary of this program. The sanitary sewage condition was assessed using a binary method, considering whether or not the household had access to the general sewage network. ENANI-2019 considered if access was to general or rainwater networks. The tap water coverage was assessed using a binary method, considering whether or not the household had access to the general distribution network. Internet access was analyzed by household, and, in the PNDS 2006, it was conditioned to having a computer.

### Indicators used to assess nutritional transition

To analyze the time trend from 2006-2019, indicators referring to micronutrient status, anthropometric status and breastfeeding practice were considered. The indicators for micronutrient status were: prevalence of anemia (hemoglobin < 11g/dL) ^20^ and vitamin A deficiency (serum retinol < 0.7μmol/L) ^21^ in children 6-59 months of age.

In PNDS 2006, the measurements of these micronutrients were performed based on capillary blood samples after the reconstitution of dry blood spot ^9^, consisting of a blood sample collection with a micro-lancet, which was then deposited on filter paper. Hemoglobin levels were evaluated by the cyanmethemoglobin method, and vitamin A levels were assessed by high-performance liquid chromatography (HPLC). In ENANI-2019, the analyses were performed using a venous blood sample ^22^. The evaluation of hemoglobin concentration was conducted in the hematology analyzer with cell analysis by flow cytometry (UniCell DxH; https://www.beckmancoulter.com/), and in the vitamin A evaluation, HPLC with ultraviolet detection (HPLC Chromsystems; https://chromsystems.com/) was used.

For children 0-59 months of age, the anthropometric assessment was performed based on the indicators of excessive weight (BAZ > 2) and stunting (HAZ < -2) ^23^. In ENANI-2019, the first measurements of weight and height (length or stature) were used to calculate the Z-score of the anthropometric indices BAZ and HAZ. For full-term children (37-42 gestational weeks at birth) or preterm (< 37 gestational weeks at birth) who were aged > 454 days from conception (calculated by the sum between gestational age at birth and postnatal age, in days), the indices were calculated as Z-scores according to the World Health Organization (WHO) standard growth curves ^23^. For children born prematurely and aged 189-454 days from conception, the HAZ index was calculated using the INTERGROWTH-21^st^ postnatal growth curve of premature infants as a reference ^19^ ^,^ ^24^. Due to the absence of a reference curve, the BAZ was not calculated for preterm infants aged < 454 days from conception.

In PNDS 2006, the first measurements of weight and height (length or stature) were used to calculate the anthropometric indices. The HAZ and BAZ were calculated according to the WHO curves ^23^. Due to the absence of information on gestational age at birth, prematurity was not considered for growth assessment ^25^.

For the assessment of breastfeeding practices, the prevalence of exclusive breastfeeding among children < 6 months and the prevalence of continued breastfeeding among children 12-23 months ^26^ were considered. Exclusive breastfeeding among children < 6 months was calculated considering the proportion of children < 6 months of age who had received breast milk exclusively on the day before the survey, considering ages of < 183 days in ENANI-2019 and < 6 months in PNDS 2006. Continued breastfeeding among children 12-23 months was defined as the proportion of children 12-23 months of age who had received breast milk on the day before the interview, considering ages of ≥ 365 and < 730 days in ENANI-2019 and ages of > 12 and < 23 months in PNDS 2006.

For the description of diet quality, it was not possible to construct comparable indicators between the two surveys, since in ENANI-2019, the questions were related to food consumption on the day before the interview and, in the PNDS 2006, they mostly referred to the week before the survey.

In ENANI-2019, a minimum dietary diversity (MDD) was defined as the consumption of at least five of the eight following food groups: breast milk; grains, roots and tubers (porridge, bread, rice, pasta, baby cereal, potatoes, other starchy vegetables); beans, nuts and seeds; dairy products (animal milk, infant formula, yogurt, porridge); flesh foods (animal meat, liver, kidney, heart, sausages, processed meats); eggs; vitamin A-rich fruits and vegetables (carrots, pumpkin, sweet potato, cabbage, spinach, other local dark green, leafy vegetables); other fruits and vegetables ^26^.

The indicator of ultra-processed food consumption considered those who ate at least one of the following products: carbonated drinks; other sugar-sweetened beverages (industrialized juice, boxed juice, boxed coconut water, guarana syrup, redcurrant soft drink, powdered juice, or natural fruit juice with added sugar); packaged snacks (including chips); sweet or salty cookies/crackers; candies (confectionery); yogurts; processed breads (flatbread, breadsticks, and hamburger buns); instant flours (rice, corn, wheat, or oatmeal); processed meats (hamburger, ham, mortadella, salami, nugget, sausage, or frankfurter); instant noodles. Indicators of fruits or vegetables nonconsumption and meat consumption (any type of meat, including viscera and ultra-processed meats) or eggs were also calculated ^26^. All dietary quality indicators were constructed for children 6-59 months of age (≥ 183 days to < 1,826 days).

### Data analysis

The analyses were performed considering the sample design of the surveys, using the R software (http://www.r-project.org) *survey* package. The Z-score values of anthropometric indices were calculated using the *who_bmi2zscore* and *who_lencm2zscore* functions of the *growthstandards* package. Prevalence and respective 95% confidence interval (95%CI) were estimated for each outcome. The difference between estimates was considered statistically significant when there was no overlap between the 95%CIs. The coefficient of variation (CV) is a measure of dispersion, indicative of the heterogeneity of the data, and it is calculated as the ratio of the standard error and estimated prevalence value. Estimates were considered accurate when CV ≤ 30% ^27^. The total population per thousand units was presented, representing children or households. In the analyses of the mothers’ race/skin color, we chose to omit “yellow” and “indigenous” due to the low precision of the indicators estimates for these subgroups, which represented approximately 5% of PNDS 2006 sample and 1% of ENANI-2019 sample.

In PNDS 2006, missing values were disregarded in the analyses. In ENANI-2019, the variables were imputed to replace implausible values and “do not know/did not want to answer” responses ^15^ ^,^ ^16^.

Equiplot charts were constructed for the six outcomes studied in the time trend analysis to examine regional inequalities (macroregions: North, Northeast, Southeast, South, and Central-West), according to maternal schooling level (0-7, 8-10, 11, or ≥ 12 years of education), as a proxy for socioeconomic level, and according to self-reported maternal race/skin color (white, mixed-race, or black), considering PNDS 2006 and ENANI-2019. The equiplot is a mean of graphic representation that allows researchers to study the absolute inequality between groups and to present the magnitude of prevalence and differences between groups ^28^. It allows them to view, at the same time, the situation of each group in relation to the indicator and the distance between groups - which expresses the dimension of inequalities: the greater the distance between the groups, the greater the inequality.

## Results

Several socioeconomic and demographic indicators improved from 2006 to 2019: maternal schooling of ≥ 11 years increased from 32.2% to 56.2%, general sewage system coverage increased from 46% to 74.8%, and access to tap water increased from 79.3% to 93.3%. Participation in the Brazilian Income Transfer Program increased from 22.9% to 35.2%, and internet access increased from 11.6% to 61.6% (Table 1).

**Table 1:** Sociodemographic indicators in the *Brazilian National Survey on Demography and Health of Women and Children* (PNDS 2006) and in the *Brazilian National Survey on Child Nutrition* (ENANI-2019).

Unit of analysis/Indicator	PNDS 2006			ENANI-2019		
	Frequency (%)	95%CI	Units (x 1,000) *	Frequency (%)	95%CI	Units (x 1,000) *
Child						
Maternal race/skin color						
White	34.2	31.6; 36.9	4,625.3	31.7	29.2; 34.2	4,678.1
Black	10.7	8.5; 13.0	1,449.3	12.7	11.0; 14.5	1,878.7
Mixed-race	49.7	46.5; 52.8	6,712.5	54.5	51.8; 57.2	8,050.9
Mother or caregiver education level (years of education) **						
0-7	40.7	37.5; 44.0	5,547.1	22.4	20.6; 24.3	3,313.0
8-10	27.0	24.2; 29.9	3,681.6	21.3	19.6; 23.1	3,148.2
11	24.9	21.8; 28.0	3,391.8	39.5	37.3; 41.7	5,830.0
≥ 12	7.3	5.7; 8.9	992.1	16.7	15.0; 18.5	2,472.7
Household						
Brazilian Income Transfer Program						
Enrolled	22.9	20.2; 25.6	2,713.9	35.2	32.3; 38.0	4,496.0
Not enrolled	77.1	74.4; 79.8	9,122.6	64.8	62.0; 67.7	8,290.6
General sewage network						
Yes	46.0	41.8; 50.2	5,425.6	74.8	71.5; 78.1	9,562.4
No	54.0	49.8; 58.2	6,361.6	25.2	21.9; 28.5	3,224.3
Pipped water						
Yes	79.3	76.0; 82.7	9,394.1	93.3	90.9; 95.6	11,924.1
No	20.7	17.3; 24.0	2,444.8	6.7	4.4; 9.1	862.5
Internet access						
Yes	11.6	9.4; 13.8	1,296.3	61.6	58.0; 65.3	7,881.9
No	88.4	86.2; 90.6	9,856.7	38.4	34.7; 42.0	4,904.8

95%CI: 95% confidence interval.

Note: estimates were calculated incorporating the surveys’ sample design and considering only the valid values.

* Indicates that the value presented in each cell of the table must be multiplied by 1,000 to obtain the total population in that condition;

** PNDS 2006 collected the maternal education and ENANI-2019 collected the education of the mother or caregiver.

The prevalence of anemia among children 6-59 months of age decreased from 20.5% to 10.1%, and vitamin A deficiency decreased from 17.2% to 6% between surveys. The prevalence of stunting among children 0-59 months of age remained around 7%, while excessive weight increased from 6% to 10.1%. The prevalence of exclusive breastfeeding among children < 6 months increased from 38.6% to 45.8%, and that of continued breastfeeding among children 12-23 months increased from 34.6% to 43.6%, although no statistically significant differences were observed.

The diet-related quality indicators among children 6-59 months of age revealed that, in 2019, 61.5% of them presented MDD, 88.8% consumed ultra-processed foods, 25.7% did not eat fruits or vegetables, and 83.1% consumed meat or egg the day before the survey (Table 2).

**Table 2:** Food and nutritional indicators in the *Brazilian National Survey on Demography and Health of Women and Children* (PNDS 2006) and in the *Brazilian National Survey on Child Nutrition* (ENANI-2019).

Indicator	PNDS 2006			ENANI-2019		
	Prevalence (%)	95%CI	Children (x 1,000) *	Prevalence (%)	95%CI	Children (x 1,000) *
Anemia	20.5	17.3; 23.6	1,910.9	10.1	8.0; 12.1	1,338.9
Vitamin A deficiency	17.2	14.4; 20.1	1,625.4	6.0	5.2; 6.9	800.9
Stunting (Z < -2)	7.3	5.9; 8.7	904.6	7.0	6.0; 7.9	1,026.2
Excessive weight (BMI, Z > 2)	6.0	4.9; 7.1	742.1	10.1	9.0; 11.1	1,473,5
Exclusive breastfeeding among children < 6 months	38.6	30.6; 46.6	587.9	45.8	40.9; 50.7	681.4
Continued breastfeeding among children 12-23 months	34.6	29.1; 40.0	890.8	43.6	39.4; 47.7	1,292.4
Minimum dietary diversity	-	-	-	61.5	58.7; 64.4	8,171.4
Consumption of ultra-processed foods	-	-	-	88.8	87.0; 90.7	11,795.9
Nonconsumption of fruits or vegetables	-	-	-	25.7	23.1; 28.3	3,409.4
Consumption of meat or egg	-	-	-	83.1	81.2; 84.9	11,030.2

95%CI: 95% confidence interval; BMI: body mass index.

* Indicates that the value presented must be multiplied by 1,000 to obtain the total population in that condition.

**Figure 1:**
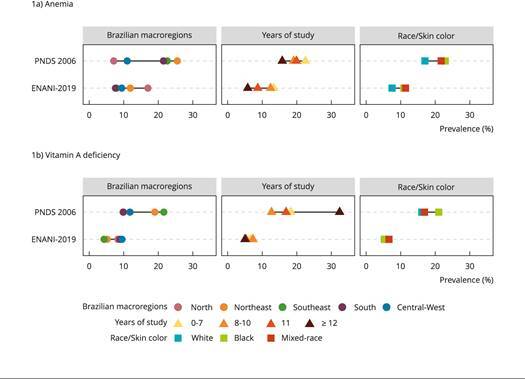
Prevalence of anemia and prevalence of vitamin A deficiency in Brazilian children 6-59 months of age in the *Brazilian National Survey on Demography and Health of Women and Children* (PNDS 2006) and in the *Brazilian National Survey on Child Nutrition* (ENANI-2019) according to sociodemographic variables *.

Note: all estimates were calculated considering the survey sample design. The schooling category of ≥ 12 in the anemia indicator of PNDS 2006 demonstrated low precision of the estimate (coefficient of variation - CV = 30.1%). * In PNDS 2006, years of study and race/skin color refer to those of the child’s mother, whereas in ENANI-2019 they refer to the child’s mother or caregiver. Yellow and indigenous race categories are not depicted in the figure.

The inequalities in anemia prevalence reduced from 2006 to 2019, mostly for geographical regions and, to a lesser extent, for maternal race/skin color; whereas for years of maternal schooling, it remained homogenous. The disparity in vitamin A deficiency prevalence decreased for all variables, but especially for years of schooling. It seems, however, inconsistent in women with ≥ 12 years of education in the PNDS 2006 data (Figure 1).

**Figure 2:**
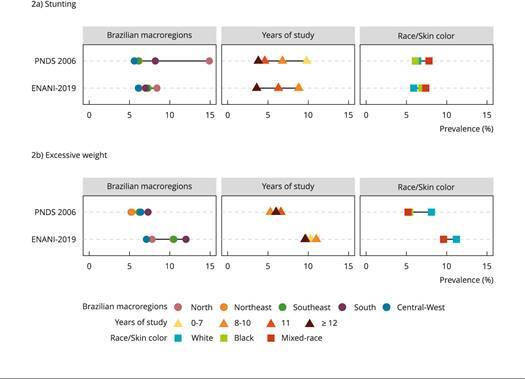
Prevalence of stunting * and excessive weight ** in Brazilian children < 59 months of age in the *Brazilian National Survey on Demography and Health of Women and Children* (PNDS 2006) and in the *Brazilian National Survey on Child Nutrition* (ENANI-2019) according to sociodemographic variables ***.

Note: prematurity was not considered for growth assessment due to the absence of information on gestational age at birth in PNDS 2006. Implausible values were excluded, as follows: body mass index (BMI)-for-age of > -5 and of < 5; height-for-age of > -6 and of < 6. All estimates were calculated considering the survey sample design. The schooling category of ≥ 12 in the stunting indicator of PNDS 2006 demonstrated low precision of the estimates (coefficient of variation - CV = 52.7%). * Z < -2 according to the height-for-age index ^17^ ^,^ ^23^; ** Z > 2 according to the BMI-for-age index ^17^ ^,^ ^23^; *** In PNDS 2006, years of study and race/skin color refer to those of the child’s mother, whereas in ENANI-2019 they refer to the child’s mother or caregiver. Yellow and indigenous race categories are not depicted in the figure.

The disparity in stunting prevalence reduced substantially from 2006 to 2009 according to geographical region and, to a lesser extent, to years of education; while for maternal race/skin color it remained similar. In contrast, disparities in excessive weight increased according to geographical region, remained similar for schooling, and were reduced for maternal race/skin color (Figure 2).

**Figure 3:**
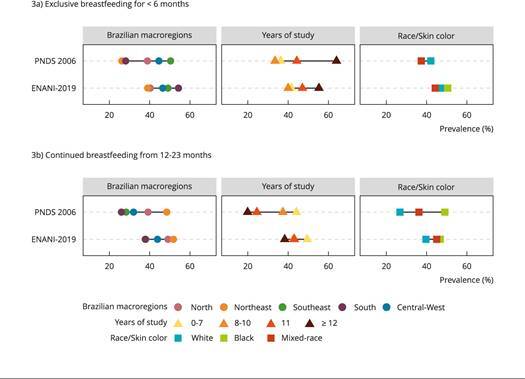
Prevalence of exclusive breastfeeding among children < 6 months and continued breastfeeding among children 12-23 months in Brazilian children 6-59 months of age in the *Brazilian National Survey on Demography and Health of Women and Children* (PNDS 2006) and in the *Brazilian National Survey on Child Nutrition* (ENANI-2019) according to sociodemographic variables *.

The disparity in exclusive breastfeeding among children < 6 months prevalence was reduced for geographical regions and years of education (partly due to the decrease in the prevalence of this practice among women with a higher schooling level). A similar situation of disparity reduction was displayed when continued breastfeeding among children 12-23 months prevalence was considered. The similarity between the prevalence observed among maternal race/skin color categories remained for exclusive breastfeeding among children < 6 months, and for continued breastfeeding among children 12-23 months, these disparities decreased (Figure 3).

* In PNDS 2006, years of study and race/skin color refer to those of the child’s mother, whereas in ENANI-2019 they refer to the child’s mother or caregiver. Yellow and indigenous race categories are not depicted in the figure. Note: all estimates were calculated considering the survey sample design. In PNDS 2006, the black race/skin color category in the exclusive breastfeeding among children < 6 months indicator and the schooling category of ≥ 12 in the continued breastfeeding among children 12-23 months indicator demonstrated low precision in the estimates (coefficient of variation - CV = 31.2% and 43.1%, respectively).

## Discussion

The temporal trends analyzed in this study revealed a decrease in micronutrient deficiencies and an increase in excessive weight and breastfeeding. Reduced regional, maternal schooling level and maternal race/skin color inequalities were observed for stunting, anemia, vitamin A deficiency, exclusive bresatfeeding among children < 6 months, and continued breastfeeding among children 12-23 months from 2006-2019. These changes can be attributed to improved living conditions (partly captured by socioeconomic and demographic indicators examined in this study) and the expansion of public health and feeding and nutrition policies implemented in 2015 ^29^ ^,^ ^30^ ^,^ ^31^. It is unknown whether these indicators were even better in 2015 and deteriorated afterward due to the recession, economic austerity measures and dismantling of public policies to guarantee rights that occurred in Brazil from 2016 onwards ^12^ ^,^ ^13^. Notably, the scenario for these indicators was better in 2019 than in 2006.

Considering the improvement in living conditions from 2003-2015 and the aforementioned public policies dismantling from 2016 onwards, we hypothesized that the stability of the stunting prevalence among children < 59 months of age might not reveal the evolution dynamics of this outcome and could be concealing the time trend of linear growth in different age groups. This indicator stability seems to result from a better growth profile among children ≥ 24 months of age combined with a worse profile among those 0-23 months of age. For children < 24 months of age, higher mean HAZ was observed in 2006 rather than in 2019, while the opposite was observed for those ≥ 24 months of age ^32^.

Among the factors that may explain the increase in excessive weight prevalence between 2006 and 2019 is the expressive consumption of ultra-processed foods, which has been reported across various realities in local Brazilian studies ^33^ ^,^ ^34^ and in ENANI-2019 itself ^35^, and has also been associated with adverse outcomes in children ^36^. Relvas et al. ^33^ observed a ultra-processed food intake of 43.1% among children 6-12 months of age assisted in primary healthcare units in a city of the São Paulo’s metropolitan region. Pereira et al. ^34^ demonstrated a high prevalence of regular ultra-processed food consumption by children 24 months of age, namely: 29.6% for instant noodles, 64.4% for sweet biscuits or sandwich cookies, 65.8% for boxed or bottled juice, powdered juice, or boxed coconut water, and 88.3% for yogurt, among other ultra-processed foods. In a systematic review on the impacts of ultra-procesed food consumption on maternal and child health, Oliveira et al. ^36^ observed that the greater participation of these foods in children’s diets has been associated with increased weight gain, adiposity measures, overweight, early weaning, poor diet quality, metabolic changes, diseases, and consumption of chemicals from materials (plastic, paper, metal, and glass) used in food packaging.

Other concerning results are the prevalence of nonconsumption of fruits or vegetables on the day before the interview (25.7%) and the prevalence of children not reaching the MDD according to ENANI-2019 (38.5%). The best result among dietary quality indicators was observed for the consumption of meat or egg (83.1%), sources of nutrients essential for linear growth, such as proteins, essential fatty acids, vitamin B12, vitamin D, zinc, phosphorus, and selenium ^35^.

We observed an increase in breastfeeding prevalence indicators from 2006-2019, however, without statistical significance. Promoting, protecting, and supporting breastfeeding are priorities in the Brazilian National Policy for Integral Child Health Care (PNAISC) ^37^. The relative stabilization of breastfeeding indicators observed may have occurred due to the limitations in the implementation of breastfeeding strategies in primary health care ^38^ and hospital settings ^39^ and to the widespread and persistent practice of abusive marketing of foods and products that directly compete with breastfeeding ^40^ ^,^ ^41^, violating national marketing regulation laws ^42^.

A reduction in regional, maternal schooling and maternal race/skin color disparities observed for stunting, anemia, vitamin A deficiency, exclusive breastfeeding among children < 6 months, and continued breastfeeding among children 12-23 months from 2006-2019 suggests that public policies that have contributed to the improvement of the indicators have also reduced the vast inequalities in Brazil. However, these inequalities are not fully mitigated, as regional and sociodemographic differences for these indicators were still observed in 2019 ^35^ ^,^ ^43^ ^,^ ^44^. For example, anemia prevalence among children aged 6-59 months decreased from 20.5% in 2006 to 10.1% in 2019, but reached 17% in the North Region, whereas it varied from 7.6% to 11.9% among other regions of Brazil ^45^. The findings regarding excessive weight converge with what has been recorded in the literature in recent years: a rise in excessive weight at increasingly earlier ages ^46^, with a more intense increase among children living in higher income and urbanization contexts ^47^, as is the case of the Southeast and South regions when compared to the others.

The main limitation of this study is the lack of full comparability between some of the PNDS 2006 and ENANI-2019 indicators due to methodological differences. These concern the diet quality, assessment of micronutrient deficiencies, and anthropometry data collection. The methods of measurement of anemia and vitamin A deficiency adopted in PNDS 2006 are not considered the gold standard. Possibly, part of the difference observed between anemia and vitamin A deficiency prevalence in 2006 and 2019 is due to methodological differences. The impossibility of considering prematurity in the PNDS 2006 could lead to an overestimation of stunting. Nonetheless, we reached the conclusion that the stunting prevalence was very similar, comparing results with and without preterm birth, similarly to INTERGROWTH-21^st^ researchers ^24^. Therefore, it is safe to assume a negligible role of this limitation on the anthropometric estimates.

As a strength of the manuscript, we highlight the methodological procedures adopted to harmonize the two studies’ databases (e.g., standardization of age calculation based on the day of anthropometric assessment and use of the first weight and height measurement to calculate anthropometric indices). Another positive aspect was the use of equiplot charts, which help visualize the evolution of inequalities over time and offer information for greater adequacy of public policies.

The evidence produced by this study points to a challenging scenario for public health. It is necessary to continue strengthening public policies and improving those aimed at promoting adequate and healthy food consumption ^48^ ^,^ ^49^ and protecting, promoting and supporting breastfeeding ^37^, which still has a prevalence below international targets, e.g., 45.8% exclusive breastfeeding among children < 6 months, while the WHO target for 2030 for this indicator is at least 70% ^50^.

Regarding anemia and vitamin A deficiency, which showed a significant decline in magnitude, the short-term challenge is the reorientation of programs based on prophylactic micronutrient supplementation aimed at preventing and controlling these diseases. The Brazilian Ministry of Health has undertaken efforts to focus on more vulnerable groups and regions and reformulate some of these programs ^51^ ^,^ ^52^.

The most effective measures to overcome stunting are: increasing the purchasing power of the poorest and universalizing the population’s access to essential education, health, and sanitation services ^53^. As mentioned, evidence indicates that the stability observed for this indicator relies on the combination of a better nutritional status among older children and a worse status among younger children ^32^. If younger children’s growth profile is maintained, there may be an increase in stunting prevalence in the near future. The effects of the COVID-19 pandemic may have exacerbated this scenario. An indication of this is the increase in the prevalence, in recent years, of food insecurity in Brazil and its intensification in the pandemic period: from 22.9% in 2013-2014 to 36.7% in 2017-2018, reaching 58.7% in 2021-2022 ^14^ ^,^ ^54^. Given this, the challenges for public policies aimed at overcoming food insecurity, hunger, and malnutrition are of different orders: mitigate the immediate effects of this pandemic and resume, improve, and expand the coverage of public policies to guarantee rights.

Regarding poor-quality diet and excessive weight, there is an urgent need to improve regulatory measures to protect children from exposure to ultra-processed foods supply and marketing communication practices, which are still incipient in Brazil ^55^. Appropriate measures for this purpose are being implemented in different countries, including regulation of advertising aimed at children, taxation of sugar-sweetened beverages, front-of-pack nutrition warning labeling of ultra-processed foods, and regulation of food environments ^56^. These measures must be articulated with actions that promote health and adequate eating habits in primary health care and schools and with initiatives that favor environments that promote physical activity ^48^ ^,^ ^49^ ^,^ ^57^. Furthermore, they must be articulated with public policies that favor access and consumption of unprocessed or minimally processed foods ^58^.

Although the actions to overcome challenges posed by the current feeding and nutritional profile of Brazilian children have been presented as responses for each specific outcome (or set of outcomes), they should articulate and complement each other. Actions that promote, protect and support breastfeeding contribute to increase this practice’s prevalence and prevent stunting and childhood obesity. Access to diversified unprocessed or minimally processed foods contributes to the prevention of obesity, stunting and micronutrient deficiencies. Actions to overcome regional and sociodemographic inequalities benefit all the outcomes addressed here. From this perspective, an additional challenge for public policymakers, researchers, and workers in feeding and nutrition is understanding that all outcomes addressed in this study are not isolated events but different faces of malnutrition ^59^ ^,^ ^60^ ^,^ ^61^.

The set of actions presented involves different public policy sectors at national, state, and municipal levels of administration. Although the health sector is the protagonist of this agenda, these actions are not limited to this sector. It is fundamental to resume the implementation of the Brazilian National Food and Nutrition Security Policy (PNSAN) and the Food and Nutrition Security System (SISAN) ^62^, which has been interrupted since 2019 ^63^, at the federal level, to enhance the intersectoral articulation that is essential for the implementation of the necessary food and nutrition policies ^64^.

ENANI-2019 collected data just before the onset of the COVID-19 pandemic, which deepened Brazil’s economic recession and social inequalities. The results of the ENANI’s second edition, which is planned for 2024, will allow us to assess this pandemic effects on the feeding and nutrition of children < 5 years old and on regional and sociodemographic inequalities regarding these events. Comparing its results with those of the ENANI-2019 will provide inputs for developing and reformulating public policies about food and nutrition that can respond to the current scenario.
